# Constituents from *Vigna vexillata* and Their Anti-Inflammatory Activity

**DOI:** 10.3390/ijms13089754

**Published:** 2012-08-06

**Authors:** Yann-Lii Leu, Tsong-Long Hwang, Ping-Chung Kuo, Kun-Pei Liou, Bow-Shin Huang, Guo-Feng Chen

**Affiliations:** 1 Graduate Institute of Natural Products, Chang Gung University, Taoyuan 333, Taiwan; E-Mails: ylleu@mail.cgu.edu.tw (Y.-L.L.); htl@mail.cgu.edu.tw (T.-L.H.); 2 Chinese Herbal Medicine Research Team, Healthy Aging Research Center, Chang Gung University, Taoyuan 333, Taiwan; 3 Department of Biotechnology, National Formosa University, Yunlin 632, Taiwan; E-Mails: pcckuoo@sunws.nfu.edu.tw (K.-P.L.); brues0923@yahoo.com.tw (B.-S.H.); 4 Department of Chemistry, National Chung-Hsing University, Taichung 402, Taiwan; E-Mail: dolphineddie@yahoo.com.tw

**Keywords:** *Vigna*, isoflavone, anti-inflammatory, superoxide anion, elastase

## Abstract

The seeds of *Vigna* genus are important food resources and there have already been many reports regarding their bioactivities. In our preliminary bioassay, the chloroform layer of methanol extracts of *V. vexillata* demonstrated significant anti-inflammatory bioactivity. Therefore, the present research is aimed to purify and identify the anti-inflammatory principles of *V. vexillata*. One new sterol (**1**) and two new isoflavones (**2**,**3**) were reported from the natural sources for the first time and their chemical structures were determined by the spectroscopic and mass spectrometric analyses. In addition, 37 known compounds were identified by comparison of their physical and spectroscopic data with those reported in the literature. Among the isolates, daidzein (**23**), abscisic acid (**25**), and quercetin (**40**) displayed the most significant inhibition of superoxide anion generation and elastase release.

## 1. Introduction

Phytochemicals from dietary and medicinal plants have been supposed as promising sources of potential anticancer agents with increasing anticancer evidences coupled with considerations of safety and efficacy [1,2]. Dietary and medicinal plants are importrant sources of phytochemicals for the treatment of cancers [3]. Several phytochemcials purified from natural plants, such as curcumin [4,5], epigallocatechin gallate [6], and soy isoflavones [7] had already been studied in various phases of clinical trials. The rapid population growth increase and inadequate supplies of foods had resulted in the nutrition deficiencies among the people living in the developing countries. The rigorous world food problem has presented an urgent condition for the nutritionists to investigate the choices of utilizing some less known crop seeds as additional sources of foods. With increasing interest in new food sources, the seeds of wild relatives of cultivated plants including the tribal pulses are now receiving more and more attention. The history of legumes is tied in closely with that of human civilization, appearing early in Asia, America, and Europe by thousand years ago, where they became essential for supplementing protein. Recently, there was a report regarding the chemical constituents in the wild soybean *Glycine soja* and their biological activity [8]. It initiated our interests to explore the chemical compositions of the wild relatives of edible legumes. For example, the seeds of *Vigna vexillata* are boiled and consumed by the tribal people living in the hilly region of Pune district, India [9,10]. The proximate composition, minerals, seed protein fractions, amino acids, fatty acids, and antinutritional factors of the seeds of *V. vexillta* were analyzed. However, information on the biochemical composition and chemical constituents of the wild relatives of grain legumes is very rare.

*V. vexillata* (L.) A. Rich (Fabaceae) is a perennial climbing or trailing herb belonging to the *Vigna* genus. This genus is widely distributed in tropical Africa, India, Indochina, Australia, Japan, Korea, China, and Taiwan. In Taiwan, the *Vigna* genus usually grows in grassland, margin of bush, at elevation between 1000 and 1800 m high, in the central mountain area [11]. The extracts of *Vigna* species have been reported to display the hypoglycemic [12,13], antihypertensive [14,15], cholesterol reduction [16], antioxidant [17,18], antibacterial [19,20], and anti-cancer bioactivities [21–23]. In the preliminary bioassay, at the tested concentration (10 μg/mL) the methanol extract, chloroform, and water fractions of *V. vexillata* displayed the inhibition of superoxide anion generation and elastase release with percentages of 22.06 ± 5.66%, 57.65 ± 3.69%, and 11.08 ± 5.19%; 19.35 ± 1.52%, 67.27 ± 3.53%, and 11.00 ± 3.37%, respectively (Table 1). Therefore, it is aimed to purify and identify the chemical constituents of the methanol extract *V. vexillata* in the present research. Furthermore, the anti-inflammatory bioactivities of these purified constituents were also examined to explore the new candidates of phytomedicinal lead compounds.

## 2. Results and Discussion

### 2.1. Purification and Characterization

Air-dried and powdered whole plants of *V. vexillata*, including flowers and pods, were extracted with methanol under reflux and concentrated to give a dark brown syrup. The methanol extracts were suspended in water, and successively fractionated with chloroform to afford chloroform and water solubles, respectively. With the assistance of a combination of conventional chromatographic techniques, one new sterol (**1**) and two new isoflavones (**2,3**) were determined by the 1D and 2D NMR elucidations, and mass spectral analyses. In addition, 37 known compounds, including stigmast-4-en-3-one (**4**), stigmast-4,22-dien-3-one (**5**) [24], (27*RS*)-cycloart-28-en-3β,27-diol (**6**) [25], β-sitosterol (**7**), stigmasterol (**8**) [26], sitosterol *cis*-*p*-coumarate (**9**) [8], sitosterol *trans*-*p*-coumarate (**10**) [8], lupeol (**11**) [26], 6β-hydroxy-β-sitosterone (**12**) [27], sitosterol ferulate (**13**) [28], (20*R*)-22*E*-cholest-4-ene-3,6-dione (**14**) [29], methylparaben (**15**) [30], *p*-hydroxybenzoic acid (**16**) [30], *p*-hydroxybenzaldehyde (**17**) [30], vanillic acid (**18**) [30], genistein (**19**) [31], dehydrovomifoliol (**20**) [32], β-sitostenone (**21**) [33], 5,7,4′-trihydroxy-3′-methoxy isoflavone (**22**) [34], daidzein (**23**) [35], indole-3-carboxaldehyde (**24**) [36], abscisic acid (**25**) [37], *trans*-cinnamic acid (**26**) [38], β-sitosteryl-3-*O*-β-glucopyranoside (**27**) [39], *trans*-methyl *p*-coumarate (**28**) [30], salicylic acid (**29**) [40], tachioside (**30**) [41], 3-hydroxy-β-damascone (**31**) [42], *p*-hydroxyl phenethanol (**32**) [43], *trans-p*-coumaric acid (**33**) [44], 3,6-dihydroxy-5,6-dihydro-β-ionol (**34**) [45], dihydrophaseic acid (**35**) [46], blumenol A (**36**) [47], isovitexin (**37**) [48], daidzin (**38**) [49], vitexin (**39**) [50], and quercetin (**40**) [51] were characterized by comparison of their physical and spectroscopic data with those reported in the literature.

### 2.2. Structural Elucidation of New Compounds **1–3**

**Figure 1:**
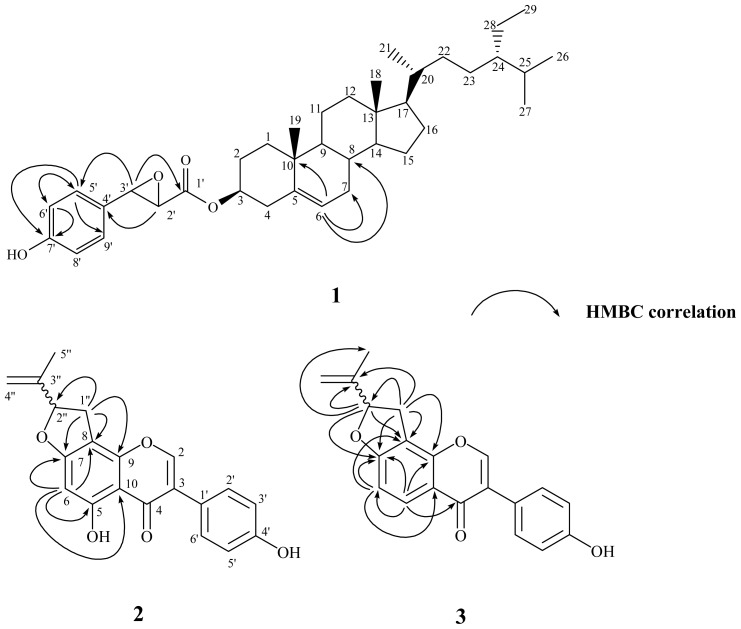
Chemical structures and significant heteronuclear multiple bond coherence (HMBC) correlations of **1**–**3**.

The purified solid **1** was visualized by spraying with 1% (w/v) Ce(SO_4_)_2_ in 10% (v/v) aqueous H_2_SO_4_ followed by heating at 120 °C and displayed purplish black spots on TLC plate. They also displayed positive responses against the Lieberman–Burchard test. These results suggested that compound **1** possessed steroid basic skeleton [52]. It was isolated as optically active white powder with mp 145–147 °C, and the molecular formula was established as C_38_H_56_O_4_ by the pseudomolecular ion peak at *m/z* 575.4093 ([M−H]^−^) in HR-ESI-MS analysis. The UV absorption maxima at 280 and 229 nm were characteristic of a benzene moiety [53]. The IR absorption bands at 3438 and 1721 cm^−1^ displayed the presence of hydroxyl and carbonyl groups, respectively. In its ^1^H-NMR spectrum, two singlets at δ 0.68 (3H, s, CH_3_-18) and 1.01 (3H, s, CH_3_-19); three doublets at δ 0.82 (3H, d, *J* = 6.9 Hz, CH_3_-26), 0.84 (3H, d, *J* = 6.9 Hz, CH_3_-27), and 0.92 (3H, d, *J* = 6.4 Hz, CH_3_-21); and one triplet at δ 0.85 (3H, t, *J* = 7.4 Hz, CH_3_-29), evidenced that this compound was a stigmastane derivative [28]. The resonances located at δ 4.66 (1H, m) and 5.36 (1H, br s, H-6) indicated that an oxygenated substitution and an olefinic functional group were presented in the stigmastane skeleton. In addition, in the aromatic region a typical set of A_2_B_2_ signals at δ 6.57 (2H, d, *J* = 8.3 Hz, H-6′, −8′) and 6.76 (2H, d, *J* = 8.3 Hz, H-5′, −9′) were attributed to a *para*-substituted aromatic ring. The ^13^C NMR spectrum of **1** also exhibited the characteristic signal for one ester carbonyl group at δ 172.0. The above-described spectroscopic characteristics of **1** were very similar to those of sitosterol *trans*-*p*-coumarate (**10**) [8]. The significant ^1^H and ^13^C NMR spectral differences between **1** and **10** were that the *trans* −COCH=CH– fragment in **10** was disappeared. Instead, there were two oxygenated proton signals at δ 3.68 (1H, d, *J* = 5.9 Hz, H-2′) and 4.23 (1H, d, *J* = 5.9 Hz, H-3′) indicating the epoxidation of the double bond and this was also confirmed by the corresponding carbon signals at δ 43.9 (C-2′) and 44.3 (C-3′) in the ^13^C NMR spectrum. In the HMBC spectral analysis (Figure 1), correlation peaks between H-6 (δ 5.36) and C-7 (δ 31.9), C-8 (δ 31.9), C-10 (δ 36.6), inferred that the double bond was located at C-5 and C-6. The presence of a *p*-hydroxyphenylglycidate moiety was deduced by the ^2^*J*, ^3^*J*-HMBC correlations through H-5′ (δ 6.76) to C-6′ (δ 115.0), C-9′ (129.1), C-7′ (154.0); H-6′ (δ 6.57) to C-5′ (δ 129.1), C-4′ (δ 131.1), C-7′ (154.0); H-3′ (δ 4.23) to C-5′ (δ 129.1), C-1′ (δ 172.0); and H-2′ (δ 3.68) to C-4′ (δ 131.1). This moiety was attached at C-3 of the stigmastane skeleton to form an ester linkage according to the chemical shift of H-3 (δ 4.66). The stereochemistry of substituent at C-3 was determined as β by the H-3 proton coupling full width at half maximum. The full assignments of ^1^H and ^13^C NMR signals were substantiated by extensive 2D NMR experiments. Therefore the chemical structure of **1** was established as shown in Figure 1 and named trivially as vignasterol A.

Vigvexin A (**2**), obtained as optically active white powder, was determined to be C_20_H_16_O_5_ from its HR-ESI-MS analytical data. The UV absorption maxima at 340, 296, 265 nm is typical of an isoflavone derivative [53]. The IR absorption bands at 3418 and 1656 cm^−1^ were in agreement with the presence of a hydroxyl and a conjugated carbonyl groups, respectively. In the ^1^H NMR spectrum, a downfield singlet at δ 13.26 exchangeable with D_2_O was assigned to be an intramolecular chelated hydroxyl group at C-5. A characteristic proton singlet δ 8.18 correlated to a carbon signal at δ 154.0 in HMQC spectrum was ascribed to H-2 of the isoflavone basic skeleton. The typical A_2_B_2_ doublets located at δ 6.90 (2H, d, *J* = 8.3 Hz, H-3′, 5′) and 7.43 (2H, d, *J* = 8.3 Hz, H-2′, 6′) indicated the presence of a *para*-substituted B-ring. In addition, one more aromatic singlet at δ 6.27 correlated with δ 104.0 (C-8), 106.5 (C-10), 164.7(C-5), and 167.4 (C-7) in HMBC spectrum suggested that only C-6 was not substituted in the A-ring. Another set of proton signals observed at δ 1.78 (3H, s, CH_3_-5″), δ 3.09 (1H, dd, *J* = 15.2, 8.6 Hz, H-1″), δ 3.51 (1H, dd, *J* = 15.2, 8.6 Hz, H-1″), δ 4.95 (1H, s, H-4″), δ 5.12 (1H, s, H-4″), and δ 5.46 (1H, t, *J* = 8.6 Hz, H-2″) and the ^2^*J*, ^3^*J*-HMBC correlation between H-1″ (δ 3.09 and 3.51) and C-2″ (δ 88.8), C-8 (δ 104.0), C-9 (δ 153.8), and C-7 (δ 167.4) revealed that one isopropenyl dihydrofuran fragment was attached at C-7 and C-8. Other carbon signals were correlated through the detailed 2D NMR experimental analyses and conclusively the structure of **2** was determined as shown. Due to the limited quantity of this purified compound obtained, the absolute configuration at C-2″ could not be determined and remains unknown.

Compound **3** was also assigned as an isoflavone derivative since it was found to possess characteristic UV and IR spectroscopic data as mentioned for **2**. The major differences in the ^1^H NMR spectrum of **3** were that the downfield chelated hydroxyl singlet at δ 13.26 and the aromatic singlet at δ 6.27 in **2** were replaced by a set of two mutually coupled doublets at δ 6.95 (1H, d, *J* = 8.6 Hz, H-6) and 8.05 (1H, d, *J* = 8.6 Hz, H-5), which indicated that C-5 and C-6 were not substituted. It was further confirmed by the HMBC correlations from H-6 (δ 6.95) to C-8 (δ 114.3), C-10 (δ 119.8), and C-7 (δ 165.7); and from H-5 (δ 8.05) to C-6 (δ 109.0), C-9 (δ 154.5), C-7 (δ 165.7), and C-4 (δ 175.8). The location of the fusion of the isopropenyl dihydrofuran ring was also determined to be at C-7 and C-8 since similar HMBC correlations from H-1″ (δ 3.23 and 3.64) to C-2″ (δ 88.7), C-8 (δ 114.3), C-3″ (δ 144.6), C-9 (δ 154.5), and C-7 (δ 165.7); and from H-2″ (δ 5.52) to C-5″ (δ 17.1), C-8 (δ 114.3), C-3″ (δ 144.6), and C-7 (δ 165.7) were observed. Therefore, the chemical structure of **3** was established as displayed in Figure 1 and named as vigvexin B according to the previous convention.

### 2.3. Anti-Inflammatory Activity

Overexpression of neutrophils had already been regarded to display significant correlations to various human diseases, such as rheumatoid arthritis, ischemia, reperfusion injury, chronic obstructive pulmonary disease, and asthma [54–58]. In response to diverse stimuli, activated neutrophils secreted a series of cytotoxins, such as superoxide anion and elastase [59]. Thus, in infected tissues and organs it was critical to maintain superoxide anion production and elastase release in physiological conditions. Nowadays only a few available agents could directly modulate neutrophil proinflammatory responses in clinical practice. Therefore, those purified compounds isolated in sufficient quantity were evaluated for inhibition of superoxide anion generation and elastase release by human neutrophils in response to FMLP/CB (Table 2) and **1**, **3**, **19**, **22**, **23**, **25**, and **40** at 10 μM concentration exhibited the inhibition percentages higher than 50%. Among those examined constituents, daidzein (**23**), abscisic acid (**25**), and quercetin (**40**) displayed the most significant inhibition of superoxide anion generation and elastase release with IC_50_ values ranged from 2.66 ± 0.85 to 5.51 ± 1.07 μM, compared with the reference compound LY294002 [60], which displayed IC_50_ of 1.38 ± 0.22 and 1.95 ± 0.35 μM towards superoxide anion generation and elastase release, respectively. In addition, diphenyleniodonium (DPI), a NADPH oxidase inhibitor, was also used as a positive control for superoxide anion generation with IC_50_ of 0.93 ± 0.52 μM. Therefore, the extracts and purified principles of *V. vexillata* have potential to be developed as new anti-inflammatory drugs or health foods.

## 3. Experimental Section

### 3.1. General

All the chemicals were purchased from Merck KGaA (Darmstadt, Germany) unless specifically indicated. Melting points of purified compounds were determined by a Fisher Scientific melting point measuring apparatus without corrections. Optical rotations were measured with the Atago AP-300 automatic polarimeter. The UV spectra were obtained on a GBC Cintra 101 UV-Vis spectrophotometer. The IR spectra were obtained on a Bruker Tensor 27 FT-IR spectrometer. The mass and high-resolution mass spectra were obtained on a VG platform electrospray mass spectrometer and a Thermo Fisher Scientific LTQ orbitrap XL mass spectrometer (San Jose, CA) operated both in the negative-ion and positive-ion modes. ^1^H- and ^13^C-NMR, COSY, NOESY, HMQC, and HMBC spectra were recorded on the Bruker AV-500 and Avance III-400 NMR spectrometers with tetramethylsilane as the internal standard. Standard pulse sequences and parameters were used for the NMR experiments and all chemical shifts were reported in parts per million (ppm, d). Column chromatography was performed on silica gels (Kieselgel 60, 70–230 mesh and 230–400 mesh, Merck KGaA). Thin layer chromatography (TLC) was conducted on precoated Kieselgel 60 F 254 plates (Merck) and the compounds were visualized by UV light or spraying with 10% (v/v) H_2_SO_4_ followed by heating at 110 °C for 10 min. High performance liquid chromatography (HPLC) was performed on a Shimadzu LC-10AT*VP* series pumping system equipped with a Shimadzu SPD-M10A*VP* diode array detector.

### 3.2. Plant Materials

The whole plants of *V. vexillata* L. A. Rich (Fabaceae) were collected in the river shores of Chingshui River in Nantou, Taiwan, in March 2006. The plant materials were authenticated by C. S. Kuoh (Department of Bioscience, National Cheng Kung University, Tainan, Taiwan). A voucher specimen (PCKuo_2006002) was deposited in the herbarium of Department of Biotechnology, National Formosa University, Yunlin, Taiwan.

### 3.3. Extraction and Isolation

The whole plants of *V. vexillata* L. (4.9 kg) were powdered and exhaustively extracted with methanol under reflux (10 L × 5 × 8 h), and the combined extracts were concentrated under reduced pressure to give a dark brown syrup (900 g). The crude extract was partitioned between chloroform and water to afford chloroform (210 g) and water extracts (690 g), respectively.

The chloroform extract was subjected to a silica gel column eluted with *n*-hexane and a step gradient of acetone (100:1 to 1:1) to afford 13 fractions as monitored by TLC. There were no constituents identified from fractions 1–4. Fraction 5 was subjected to silica gel column chromatography with mixture of *n*-hexane and acetone (50:1) to yield a mixture of **4** and **5** (3.0 mg), and **6** (1.5 mg). Fraction 6 was further resolved on a silica gel column eluted with *n*-hexane and a step gradient of ethyl acetate (100:1 to 1:1) to give three subfractions (6.1–6.3). Subfraction 6.1 was recrystallized with chloroform and methanol to afford a mixture of **7** and **8** (900.0 mg). Subfraction 6.2 was purified with silica gel column chromatography eluted with the solvent mixture of *n*-hexane and ethyl acetate (8:1) to yield **9** (8.2 mg), **10** (10.0 mg), and **11** (4.0 mg). Subfraction 6.3 was separated through silica gel column chromatography eluted with chloroform and methanol (100:1) and further purified with preparative TLC on silica gel to afford **12** (1.0 mg). Column chromatography over silica gel of the seventh fraction by the mixture of benzene and ethyl acetate (8:1) and followed by purification with preparative TLC on silica gel to result in **13** (3.0 mg) and **14** (0.5 mg). Fractions 8 and 9 were combined and further separated by repeated column chromatography over silica gel eluted with chloroform and a step gradient with acetone (300:1 to 1:1) followed by purification with preparative TLC on silica gel to yield **1** (2.6 mg), **15** (1.0 mg), **16** (0.5 mg), and **17** (3.0 mg). The tenth fraction was separated by silica gel column chromatography eluted with benzene and a step gradient with ethyl acetate (100:1 to 1:1) to afford six subfractions (10.1–10.6). Subfraction 10.1 was purified with preparative TLC by chloroform and acetone (300:1) to result in **18** (1.0 mg). Subfraction 10.2 was subjected into preparative TLC eluted by benzene and ethyl acetate (10:1) to afford **2** (2.5 mg). With similar procedures eluted with the solvent mixtures of chloroform and acetone (10:1), subfraction 10.3 was purified and resulted in **3** (6.0 mg). The fourth subfraction 10.4 was purified with preparative TLC by *n*-hexane and acetone (2:1) to result in **19** (3.0 mg). Subfractions 10.5 and 10.6 were resolved with preparative TLC by *n*-hexane and ethyl acetate (2:1) to afford **20** (3.0 mg) and **21** (1.5 mg), respectively. Fraction 11 was subjected into silica gel column chromatography with the mixing solvent of chloroform and a step gradient with methanol (100:1 to 1:1), and the resulted subfractions were purified with preparative silica gel TLC by chloroform and acetone (100:1) to yield **22** (2.0 mg), **23** (2.0 mg), and **24** (1.0 mg), respectively. Fraction 12 was separated by silica gel column chromatography eluted with chloroform and a step gradient with methanol (30:1 to 1:1) to afford four subfractions (12.1–12.4). There were no significant spots in subfractions 12.1 and 12.2 as monitored by TLC and thus no principles were identified from these fractions. Subfraction 12.3 was purified with the aid of preparative TLC by *n*-hexane and ethyl acetate (2:1) to yield **25** (3.0 mg) and **26** (4.0 mg). With the similar procedures, **27** (20.0 mg) was characterized from the subfraction 12.4. The last fraction of chloroform extract was separated by silica gel column chromatography eluted with chloroform and methanol (20:1) to yield **16** (0.5 mg).

The water extract was applied to a reversed-phase Diaion HP-20 column eluted with water and methanol gradients to afford 14 fractions as monitored by C-18 TLC, however, no constituents were identified from fractions 1–3. Fractions 4 and 5 were combined and subjected into C-18 column chromatography eluted with water and methanol gradients and further recrystallization with chloroform-methanol to yield **28** (6.0 mg), **29** (2.5 mg), and **30** (5.0 mg), respectively. Fractions 6–8 were merged and purified by C-18 column chromatography eluted with water and methanol gradients followed by preparative TLC eluted with chloroform and methanol (20:1) on the resulted subfractions to afford **31** (20.0 mg), **32** (1.5 mg), **33** (2.0 mg), and **34** (15.0 mg), respectively. Fractions 7–9 were merged and purified by silica gel column chromatography eluted with chloroform and methanol (50:1) and further recrystallization of methanol of the subfractions to result in **35** (3.0 mg) and **36** (2.0 mg). Fractions 12 and 13 were combined and separated by silica gel column chromatography eluted with chloroform and methanol (50:1) to afford four subfractions (12.1–12.4). Subfraction 12.1 was recrystallized with chloroform and methanol to yield **35** (2.0 mg). Subfraction 12.2 was purified by reversed-phase HPLC with a Supelco Discovery^®^ HS C-18 (250 × 4.6 mm, 5μm) column eluted with 0.5 mL/min of MeOH-H_2_O (40:60) to give **37** (5.0 mg) and **38** (4.0 mg). Subfraction 12.3 was recrystallized with chloroform and methanol to yield **39** (6.0 mg). Fraction 14 was resolved by silica gel column chromatography eluted with chloroform and methanol (50:1) and further recrystallization of methanol of the subfraction to result in **40** (30.0 mg).

#### 3.3.1. Vignasterol A (1)

White powder, mp 145–147 °C (CHCl_3_); [α]_D_^25^ −300.0 (*c* 0.1, CHCl_3_). UV (MeOH) *λ*_max_ (log ɛ): 280 (2.29), 229 (2.72, sh) nm. IR (Neat) *ν*_max_: 3438, 2956, 2868, 1721, 1642, 1516, 1461, 1377, 1261, 1207, 1172, 1103, 1063, 1024 cm^−1^. ESI-MS (*rel. int. %*): *m/z* 575 ([M−H]^−^, 47), 559 (100). HR-ESI-MS: *m/z* 575.4093 [M−H]^−^ (calcd for C_38_H_55_O_4_, 575.4095). ^1^H-NMR (CDCl_3_, 500 MHz): δ 6.76 (2H, d, *J* = 8.3 Hz, H-5′, −9′), 6.57 (2H, d, *J* = 8.3 Hz, H-6′, −8′), 5.36 (1H, br s, H-6), 4.66 (1H, m, H-3), 4.23 (1H, d, *J* = 5.9 Hz, H-3′), 3.68 (1H, d, *J* = 5.9 Hz, H-2′), 2.34 (2H, m, H-4), 2.00 (2H, m, H-7, −12), 1.85–1.87 (2H, m, H-1, −16), 1.70 (1H, m, H-25), 1.57 (3H, m, H-2, −7, −15), 1.46 (2H, m, H-8, −11), 1.36 (2H, m, H-20, −22), 1.26 (2H, m, H-16, −28), 1.18 (2H, m, H-12, −23), 1.07 (1H, m, H-1), 1.01 (1H, m, H-22), 1.01 (3H, s, CH_3_-19), 0.92 (3H, d, *J* = 6.4 Hz, CH_3_-21), 0.85 (3H, t, *J* = 7.4 Hz, CH_3_-29), 0.84 (3H, d, *J* = 6.9 Hz, CH_3_-27), 0.83 (1H, m, H-28), 0.82 (3H, d, *J* = 6.9 Hz, CH_3_-26), 0.68 (3H, s, CH_3_-18). ^13^C-NMR (CDCl_3_, 100 MHz): δ 172.0 (C-1′), 154.0 (C-7′), 139.6 (C-5), 131.1 (C-4′), 129.1 (C-5′, −9′), 122.8 (C-6), 115.0 (C-6′, −8′), 74.7 (C-3), 56.7 (C-14), 56.1 (C-17), 50.0 (C-9), 45.9 (C-24), 44.3 (C-3′), 43.9 (C-2′), 42.3 (C-13), 39.7 (C-12), 38.1 (C-4), 37.0 (C-1), 36.6 (C-10), 36.2 (C-20), 34.0 (C-22), 31.9 (C-7), 31.9 (C-8), 29.2 (C-25), 28.2 (C-16), 27.8 (C-2), 26.2 (C-23), 24.3 (C-15), 23.1 (C-28), 21.0 (C-11), 19.8 (C-26), 19.3 (C-19), 19.0 (C-27), 18.8 (C-21), 12.0 (C-29), 11.9 (C-18).

#### 3.3.2. Vigvexin A (2)

White powder, mp > 300 °C (MeOH); [α]_D_^25^ −100.0 (*c* 0.2, MeOH). UV (MeOH) *λ*_max_ (log ɛ): 340 (1.86), 296 (sh, 2.28), 265 (2.84), 222 (sh, 2.52), 213 (2.53) nm. IR (Neat) *ν*_max_: 3418, 2928, 1656, 1614, 1515, 1480, 1433, 1402, 1320, 1286, 1251, 1205, 1169, 1131, 1065, 1027 cm^−1^. ESI-MS (*rel. int. %*): *m/z* 335 ([M−H]^−^, 100), 321 (76), 305 (88). HR-ESI-MS: *m/z* 335.0905 [M−H]^−^ (calcd for C_20_H_15_O_5_, 335.0914). ^1^H-NMR (Acetone-*d*_6_, 400 MHz): δ 13.26 (1H, s, OH), 8.77 (1H, s, OH), 8.18 (1H, s, H-2), 7.43 (2H, d, *J* = 8.3 Hz, H-2′, −6′), 6.90 (2H, d, *J* = 8.3 Hz, H-3′, −5′), 6.27 (1H, s, H-6), 5.46 (1H, t, *J* = 8.6 Hz, H-2″), 5.12 (1H, s, H-4″), 4.95 (1H, s, H-4″), 3.51 (1H, dd, *J* = 15.2, 8.6 Hz, H-1″), 3.09 (1H, dd, *J* = 15.2, 8.6 Hz, H-1″), 1.78 (3H, s, CH_3_-5″). ^13^C-NMR (Acetone-*d*_6_, 100 MHz): δ 181.8 (C-4), 167.4 (C-7), 164.7 (C-5), 158.6 (C-4′), 154.0 (C-2), 153.8 (C-9), 144.6 (C-3″), 131.3 (C-2′, −6′), 124.2 (C-3), 122.9 (C-1′), 116.0 (C-3′, −5′), 112.9 (C-4″), 106.5 (C-10), 104.0 (C-8), 94.5 (C-6), 88.8 (C-2″), 31.3 (C-1″), 17.1 (C-5″).

#### 3.3.3. Vigvexin B (3)

White plates, mp 227–229 °C (MeOH); [α]_D_^25^ −80.0 (*c* 0.2, MeOH). UV (MeOH) *λ*_max_ (log ɛ): 307 (2.25), 250 (2.71), 243 (sh, 2.67), 216 (2.38) nm. IR (Neat) *ν*_max_: 3292, 2928, 2858, 1627, 1594, 1515, 1449, 1389, 1318, 1270, 1208, 1175, 1105, 1066, 1021 cm^-1^. ESI-MS (*rel. int. %*): *m/z* 343 ([M+Na]^+^, 28), 321 ([M+H]^+^, 100). HR-ESI-MS: *m/z* 343.0944 [M+Na]^+^ (calcd for C_20_H_16_O_4_Na, 343.0946), 321.1128 [M+H]^+^ (calcd for C_20_H_17_O_4_, 321.1127). ^1^H-NMR (Acetone-*d*_6_, 400 MHz): δ 8.17 (1H, s, H-2), 8.05 (1H, d, *J* = 8.6 Hz, H-5), 7.46 (2H, d, *J* = 8.6 Hz, H-2′, −6′), 6.95 (1H, d, *J* = 8.6 Hz, H-6), 6.88 (2H, d, *J* = 8.6 Hz, H-3′, −5′), 5.52 (1H, t, *J* = 7.8 Hz, H-2″), 5.15 (1H, s, H-4″), 4.97 (1H, s, H-4″), 3.64 (1H, dd, *J* = 16.0, 7.8 Hz, H-1″), 3.23 (1H, dd, *J* = 16.0, 7.8 Hz, H-1″), 1.81 (3H, s, CH_3_-5″). ^13^C-NMR (Acetone-*d*_6_, 100 MHz): δ 175.8 (C-4), 165.7 (C-7), 158.3 (C-4′), 154.5 (C-9), 153.0 (C-2), 144.6 (C-3″), 131.2 (C-2′, −6′), 128.4 (C-5), 125.3 (C-3), 124.3 (C-1′), 119.8 (C-10), 115.9 (C-3′, −5′), 114.3 (C-8), 112.8 (C-4″), 109.0 (C-6), 88.7 (C-2″), 32.0 (C-1″), 17.1 (C-5″).

### 3.4. Anti-Inflammatory Activity

#### 3.4.1. Preparation of Human Neutrophils

Neutrophils were isolated with a standard method of dextran sedimentation prior to centrifugation in a Ficoll Hypaque gradient and hypotonic lysis of erythrocytes. Blood was drawn from healthy human donors (20–30 years old) by venipuncture into heparin-coated vacutainer tubes, using a protocol approved by the institutional review board at Chang Gung Memorial Hospital. Blood samples were mixed gently with an equal volume of 3% dextran solution. The leukocyte-rich plasma was collected after sedimentation of the red cells for 30 min at room temperature. The leukocyte-rich plasma was transferred on top of 20 mL Ficoll solution (1.077 g/mL) and spun down at 400 g for 40 min at 20 °C. The granulocyte/erythrocyte pellets were resuspended in ice-cold 0.2% NaCl to lyse erythrocytes. After 30 sec, the same volume of 1.6% NaCl solution was added to reconstitute the isotonic condition. Purified neutrophils were pelleted and then resuspended in a calcium (Ca^2+^)-free Hank’s balanced salt solution (HBSS) buffer at pH 7.4, and were maintained at 4 °C before use.

#### 3.4.2. Measurement of Superoxide Anion Generation

The assay of the generation of superoxide anion was based on the SOD-inhibitable reduction of ferricytochrome c [59]. In brief, after supplementation with 0.5 mg/mL ferricytochrome c and 1 mM Ca^2+^, neutrophils (6 × 10^5^ cells/mL) were equilibrated at 37 °C for 2 min and incubated with drugs or an equal volume of vehicle (0.1% DMSO, negative control) for 5 min. Cells were activated with 100 nM FMLP during the preincubation of 1 μg/mL cytochalasin B (FMLP/CB) for 3 min. Changes in the absorbance with a reduction in ferricytochrome c at 550 nm were continuously monitored in a double-beam, six-cell positioner spectrophotometer with constant stirring (Hitachi U-3010, Tokyo, Japan). Calculations were based on differences in the reactions with and without SOD (100 U/mL) divided by the extinction coefficient for the reduction of ferricytochrome c (ɛ = 21.1/mM/10 mm).

#### 3.4.3. Measurement of Elastase Release

Degranulation of azurophilic granules was determined by elastase release as described previously [59]. Experiments were performed using MeO-Suc-Ala-Ala-Pro-Val-*p*-nitroanilide as the elastase substrate. Briefly, after supplementation with MeO-Suc-Ala-Ala-Pro-Val-*p*-nitroanilide (100 μM), neutrophils (6 × 10^5^/mL) were equilibrated at 37 °C for 2 min and incubated with drugs or an equal volume of vehicle (0.1% DMSO, negative control) for 5 min. Cells were activated by 100 nM FMLP and 0.5 μg/mL cytochalasin B, and changes in absorbance at 405 nm were continuously monitored to assay elastase release. The results were expressed as the percent of elastase release in the FMLP/CB-activated, drug-free control system.

### 3.5. Statistical Analysis

Results were expressed as mean ± S.D. Computation of 50% inhibitory concentration (IC_50_) was computer-assisted (PHARM/PCS v.4.2). Statistical comparisons were made between groups using Student’s *t* test. Values of P less than 0.05 were considered to be statistically significant.

## 4. Conclusions

The present investigation on the methanol extracts of *V. vexillata* resulted in the isolation and characterization of totally three new principles (**1–3**) along with 37 known constituents (**4–40**). In the screening of their bioactivity, three compounds, daidzein (**23**), abscisic acid (**25**), and quercetin (**40**) from the active chloroform fraction demonstrated significant anti-inflammatory potentials compared with the PI3K inhibitor LY294002 and a NADPH oxidase inhibitor DPI. It is well known that the phosphatidylinositol-3-kinase (PI3K)/protein kinase B (AKT) pathway plays an important role in neutrophil activation. The NADPH oxidase is also important in the inflammatory mechanism. Therefore, the purified principles of *V. vexillata* were potential to be developed as new anti-inflammatory drugs through the inhibition of PI3K or NADPH oxidase. This is the first report of complete chemical compositions of *V. vexillata* and it would provide the comprehensive knowledge related to the further discovery of phytochemical lead compounds from natural food sources.

## Figures and Tables

**Table 1:** Inhibitory effects of crude extract and partial purified fractions of *V. vexillata* on superoxide anion generation and elastase release by human neutrophils in response to *N*-formyl-l-methionyl-phenylalanine/cytochalasin B (FMLP/CB).

Samples	Inhibition Percentage (%) a	

	Superoxide Anion Generation	Elastase Release
methanol extract	22.06 ± 5.66 *	19.35 ± 1.52 ***
chloroform fraction	57.65 ± 3.69 ***	67.27 ± 3.53 ***
water fraction	11.08 ± 5.19	11.00 ± 3.37 *

a Percentage of inhibition (Inh %) at 10 μg/mL concentration. Results are presented as mean ± SD. (*n* = 3);

* *p* < 0.05 and

*** *p* < 0.001 compared with the control value.

**Table 2:** Inhibitory effects of purified samples from *V. vexillata* on superoxide anion generation and elastase release by human neutrophils in response to FMLP/CB.

Compounds	IC_50_ (μM) a or (Inh %) b	

	Superoxide Anion Generation	Elastase Release
**1**	(12.58 ± 0.82) ***	8.93 ± 1.64
**2**	(40.57 ± 4.06) ***	(17.27 ± 4.19) *
**3**	4.05 ± 0.66	(12.62 ± 7.17)
**9**	(6.13 ± 3.26)	(−21.93 ± 1.80) ***
**10**	(2.27 ± 2.70)	(−10.96 ± 5.47)
**13**	(15.45 ± 1.17) ***	(11.59 ± 4.53)
**19**	1.30 ± 0.27	(42.15 ± 2.88) ***
**20**	(19.26 ± 5.37) *	(11.39 ± 4.98)
**22**	5.87 ± 0.50	(19.37 ± 4.16) **
**23**	3.13 ± 0.27	4.29 ± 0.49
**25**	2.66 ± 0.85	2.71 ± 0.25
**27**	(−1.35 ± 3.50)	(16.39 ± 2.85) **
**30**	(37.34 ± 3.26) ***	(7.68 ± 5.60)
**31**	(20.28 ± 4.96) *	(20.11 ± 2.84) **
**34**	(26.38 ± 6.94) *	(31.49 ± 5.00) **
**35**	(26.81 ± 6.19) *	(24.43 ± 4.42) **
**36**	(17.15 ± 3.77) *	(17.88 ± 1.56) ***
**37**	(8.97 ± 2.64) *	(4.34 ± 0.58) **
**38**	(−0.88 ± 2.98)	(−1.04 ± 7.31)
**39**	(12.45 ± 5.61)	(−1.30 ± 3.61)
**40**	4.47 ± 0.76	5.51 ± 1.07
**LY294002** c	1.38 ± 0.22	1.95 ± 0.35
**DPI** d	0.93 ± 0.52	–

a Concentration necessary for 50% inhibition;

b Percentage of inhibition (Inh %) at 10 μM concentration. Results are presented as mean ± S.D. (n = 3–4).

* *p* < 0.05;

** *p* < 0.01;

*** *p* < 0.001 compared with the control value;

c A phosphatidylinositol-3-kinase inhibitor was used as a positive control for superoxide anion generation and elastase release;

d A NADPH oxidase inhibitor was used as a positive control for superoxide anion generation.
